# Identification of Influenza Cases During the H1N1 Pandemic in Massachusetts Using Population-Based Hospital Discharge Data

**DOI:** 10.1371/currents.RRN1256

**Published:** 2011-08-14

**Authors:** Hilary Placzek, Larry Madoff

**Affiliations:** ^*^Doctoral Candidate in Clinical and Population Health Research at UMass Medical School and ^†^Division of Infectious Diseases and Immunology, University of Massachusetts Medical School. Division of Epidemiology and Immunization, Massachusetts Department of Public Health. ProMED-mail, International Society for Infectious Diseases

## Abstract

Objectives

(1) To characterize the epidemiology of H1N1-related hospitalizations in Massachusetts; and (2) to compare characteristics of those hospitalized during periods of seasonal influenza activity and during the H1N1 pandemic.

Methods

Authors applied maximum and minimum criteria to the Massachusetts Hospital Discharge Database to identify H1N1-related hospitalizations. They constructed annual line graphs describing mean frequencies of influenza-like illness(ILI)-related discharges between 2005-2008, and compared these rates to early waves of H1N1 in 2009.

Results

During spring and summer 2009, there were significantly higher rates of ILI-related hospital discharges in Massachusetts compared to 2005-2008. Out of 359,344 total discharges between April 26-September 30,2009, H1N1-related hospitalizations ranged from 601 to 10,967 cases. Minimum criteria confirmed that H1N1 affected a younger population (50% were <18 years), with higher rates among African-Americans (18%) and Hispanics (23%) and higher rates of ICU admission (21%) compared to seasonal influenza (39%, 10%, 14%, and 17% respectively).

Conclusions

This is the first population-based assessment of epidemiological characteristics of hospitalized H1N1 cases in Massachusetts, and it is the first to include all possible hospitalized cases in the analysis. The authors confirm that large administrative data sets can detect hospitalizations for influenza during a pandemic, but estimated case counts vary widely depending on selection criteria used. Maximum criteria overestimated H1N1 activity, and those meeting minimum criteria resemble published accounts of H1N1-related hospitalizations closely.

               BACKGROUND 

      The emergence and spread of the novel influenza A (H1N1) virus resulted in extraordinary influenza activity across the United States throughout the spring, summer and fall of 2009 [1]. Previous studies assessed fatality rates and rates of healthcare utilization due to H1N1 influenza [2] [3]. However, accuracy and severity of in-hospital H1N1 case ascertainment are difficult to determine [4], and the impact of influenza-related hospitalizations and mortality are poorly quantified as a result of limitations and methodological challenges related to influenza reporting, diagnosis, and surveillance. Serological samples are considered the gold standard for influenza case identification [5], but they are rarely done during routine practice, and more importantly, reliable measures of confirmed influenza in population-based data sources have not been described. Under-reporting of hospitalizations and deaths occurs as well since not all patients undergo virologic testing. Thus, there is an inability to identify, test, confirm, and report many cases.

      Current statewide data describing hospitalized H1N1 cases in Massachusetts cannot be assessed for accuracy or completeness. Most hospitalizations and about 90% of deaths attributable to seasonal influenza are categorized as respiratory or circulatory (i.e., due to heart attack or stroke and not including the more specific diagnoses of pneumonia and influenza) but are nonetheless likely initially caused by influenza infection [6]. Mandated by CDC during the H1N1 pandemic, all hospitalized confirmed H1N1 cases are reportable to state health departments. Although all hospitalized cases with H1N1 should have been reported to the Massachusetts Department of Public Health (MDPH), this reporting is considered under-representative of the actual number of cases, and many (if not most) case reports contain incomplete information. One study confirmed the ‘undercount’ theory by using data from laboratory-confirmed cases reported to the CDC to estimate the prevalence of H1N1 cases. Correcting for under-ascertainment, the study estimated that every confirmed case of 2009 H1N1 reported from April - July represented 79 actual cases, and every hospitalized case reported may have represented an average of 2.7 total hospitalized people [7]. 

      Reliable estimates of influenza cases are important to better understand the impact of the virus on the population, how providers and healthcare facilities were affected by the epidemic, and to know costs associated with treatments and diagnoses. Many researchers turn to large administrative databases to calculate population-based measures of severity of influenza. These data sources characterize hospital encounter information through billing, diagnostic codes, and/or ICD-9 codes. Attempts to identify influenza cases in administrative datasets have been described in the literature [8] [9] [10] [11] [12], but more information is required about accurate case identification methods since currently no ‘gold standard’ influenza case selection criteria exists.

      Epidemiological assessments have compared severity of disease between patients hospitalized with seasonal influenza and H1N1. One study in a pediatric cohort found that H1N1 was associated with more severe respiratory disease than seasonal influenza [13]. Many of these studies were limited to single-center or regional assessments, however, and there are no population-based studies assessing this relationship, although reviews have described H1N1 activity in the Northern Hemisphere [14] and Southern Hemisphere [15]. 

      There are also confirmed diagnosis data suggesting that specific communities and populations, racial and ethnic groups [16] and specific age groups in particular, may be at increased risk of developing severe infection from H1N1, but this has not been examined in population-based studies using hospitalization discharge data from Massachusetts. 

      This study used the Hospital Discharge Database (HDD), which is a patient-linked listing of all hospital discharges in Massachusetts and includes up to 15 ICD-9-coded discharge diagnoses for each patient. We evaluated ICD-9 codes that correspond with common and relatively serious respiratory infections and influenza using two influenza case selection criteria to identify those hospitalized with influenza during the first wave of the H1N1 pandemic. 

      Taking into account the data and limitations addressed above, the main objectives for this study include: (1) to characterize the epidemiology of H1N1-related hospitalizations in Massachusetts; and (2) to compare characteristics of those hospitalized in Massachusetts during periods of seasonal influenza activity and during the H1N1 pandemic period.  

## MATERIALS AND METHODS

### **Data source**

      The HDD contains discharge data for all inpatients discharged from all 76 acute care hospitals in Massachusetts. This data source contains comprehensive information including socio-demographic data, clinical data, and charge data, with a total of 377 variables [17]. Data included all 351 cities and towns. For this study, patients met the following inclusion criteria: 1) discharge from any acute care hospital in Massachusetts in the time period October 1, 2004-Sept 30, 2010; and 2) assigned one or more diagnosis codes corresponding to a grouping of ICD-9 codes used to identify ILI. 

### **Time period selection**

   Confirmed-case laboratory specimen data from the Massachusetts State Laboratory indicated that between April 19-October 1, 2009, 99% of influenza virus isolates were H1N1. In addition, 34% of all submitted specimens were positive for H1N1 [18]. This time interval occurred before H1N1 vaccine was released, therefore it is a unique time to study the impact of the virus prior to the availability of immunization. Prior to April 26, 2009, seasonal influenza activity was common in Massachusetts: April 26 marks the first date that H1N1 was detected, and September 30 marks the end of non-influenza season. For comparison purposes, we applied the same selection criteria for influenza-like illness to the five prior influenza seasons between October 1, 2004-April 25, 2009. 

### **Study population identification**

   Patients assigned one or more ICD-9 diagnosis codes corresponding to specific case selection criteria were included in the analysis. Nationwide U.S. data indicated that only 7% of H1N1-related hospitalizations occurred in those ≥65 [19], so those ≥65 years of age were excluded from the study population to minimize misclassification bias introduced by non-influenza cases. We created a ‘maximum-minimum’ approach to describe influenza for case selection in this study and provide an interval within which the true frequency of cases is found. This approach includes two sets of selection criteria to characterize influenza and select cases using ICD-9 codes: (1) the ‘**maximum’** criterion applied the broadest definition of influenza. This list of codes has been validated against virologic results in a study evaluating code-based syndromic surveillance for ILI which found that 14 codes correlated highly with positive viral specimens [20]. These ICD-9 codes include codes for influenza, fever, and other respiratory illnesses (Table 1), and is currently in use in other H1N1 surveillance work using administrative data [21]. (2) The ‘**minimum’** criterion is the narrowest definition for influenza and included those patients who were assigned codes corresponding to influenza- specific diagnoses (Table 1). Prior studies identifying influenza cases in administrative datasets have used influenza-only diagnoses in addition to pneumonia codes [22].  

**Table 1: Specific ICD-9 Codes Included in Case Selection Criteria**

**Table:** 

**ICD-9 Code**	**Maximum Selection Criteria**	**Minimum Selection Criteria**
**79.99 Viral infection NOS **	**✔**	** **
**780.6 Fever**	**✔**	** **
**466 Laryngopharyngitis, acute**	**✔**	** **
**486 Pneumonia, organism NOS**	**✔**	** **
**465.9 Infectious upper resp, mult sites**	**✔**	** **
**461.9 Acute sinusitis, unspecified**	**✔**	** **
**382.9 Otitis media NOS**	**✔**	** **
**460 Nasopharyngitis, acute**	**✔**	** **
**490 Bronchitis NOS**	**✔**	** **
**786.2 Cough**	**✔**	** **
**487.1 Influenza with resp manif**	**✔**	**✔**
**487.8 Influenza with manif NEC **	**✔**	**✔**
**487 Influenza **	**✔**	**✔**
**465.8 Infectious upper resp, mult sites**	**✔**	**✔**
**488.1 H1N1 Influenza **	**✔**	**✔**

### **Statistical analyses**

   We constructed line graphs describing weekly ILI discharge rates, and calculated mean frequencies of ILI discharges corresponding to the fiscal year: MMWR Week 40 (10/1) through week 39 (9/30) to describe annual trends.  To describe variability related to ILI discharges prior to the H1N1 pandemic, we calculated mean weekly frequencies of ILI-related discharges for the period October 1, 2004-April 25, 2009, and 95% confidence intervals (CIs). We used SAS v.9.2 to conduct all analyses.

      To determine how and if the H1N1 population differed from the seasonal influenza population we compared periods of H1N1-specific influenza activity (April-Sept 2009) to those of seasonal influenza. To do this, we analyzed frequencies of ILI discharges from 2004-2009, described characteristics of patients with H1N1 and seasonal influenza, and used chi-square tests of proportions between groups to determine significance (Table 2).Those with *P*<0.05 indicate that proportions of the seasonal influenza and H1N1 influenza groups represent statistically different distributions.  

## RESULTS

### **Characterization of H1N1-related hospitalizations in Massachusetts**

For fiscal year 2009 (October 1, 2008 –September 30, 2009) there were 853,446 total discharges. Out of 359,344 total discharges between April 26-September 30, 2009, there were 10,976 individuals discharged from an acute care hospital in Massachusetts meeting the maximum selection criteria (Table 2) during the H1N1 pandemic. Between October 1, 2004-April 25, 2009, there were 109,084 individuals discharged with an ILI diagnosis code corresponding to influenza. According to the minimum criteria, there were 601 individuals discharged during the H1N1 pandemic (Table 2), and 2,715 patients discharged from a Massachusetts hospital between October 1, 2004-April 25, 2009.

****

**Figure:**
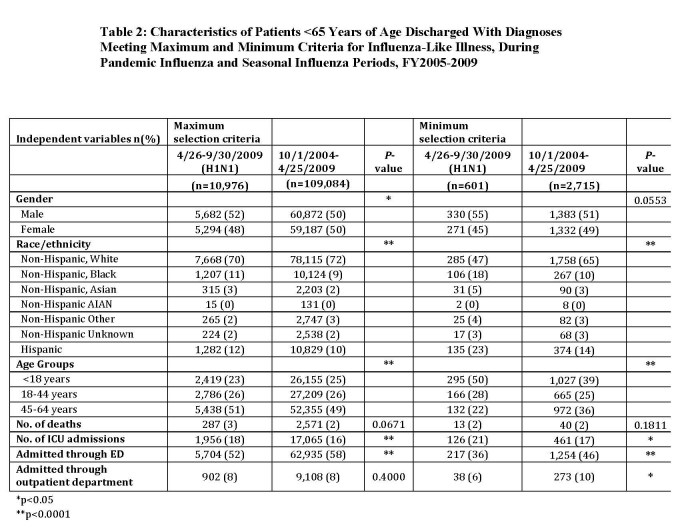

      In general, there were more narrow confidence intervals during periods of relatively low ILI activity (Weeks 40-46, or 21-27) (Figure 1).  

**Figure:**
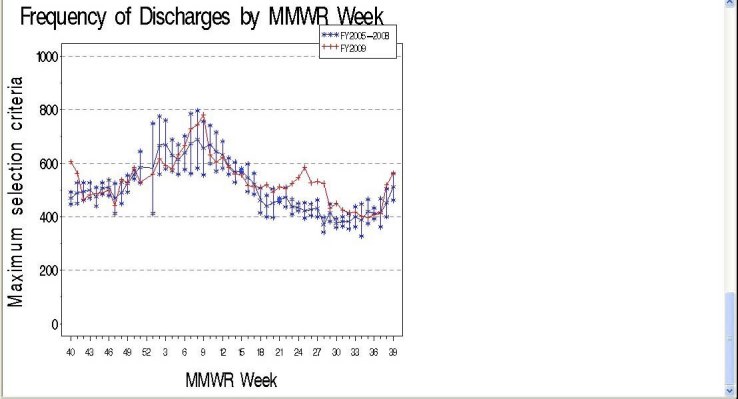
**Figure 1: Frequency Meeting Maximum Criteria in Massachusetts by MMWR Week, FY2005-200**

Legend: Figures 1 and 2 show the mean frequency of ILI discharges by MMWR week for fiscal years 2005-2008 (blue) and 2009 (red) with 95% CI’s.  

Except for Week 9, ILI discharges in 2009 fell within the 95% CI for 2005-2008 until Week 17. Starting at Week 17, however, there was an unusual increase in frequency of ILI discharges corresponding to the H1N1 pandemic. During weeks with low activity during fiscal years 2005-2008, there was a baseline of 400-500 discharges, with peaks at approximately 700 cases during Weeks 3 and 9. Activity during 2009 peaked at Week 10 with 800 discharges and then at week 25 with approximately 650 cases, compared to approximately 425 for 2005-2008. This comparison indicates typical ILI activity during fall 2008 and winter 2009, but unusually high frequency of ILI discharges during spring and summer 2009.

      Influenza-specific codes are rarely used in summer months identifying approximately zero cases except for Summer 2009 (Figure 2).  

**Figure:**
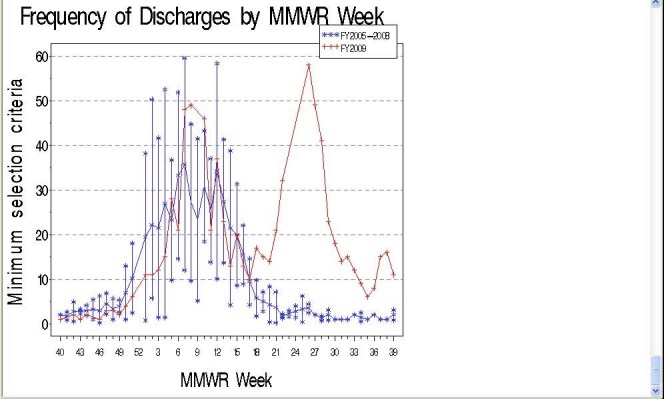
**Figure 2: Frequency Meeting Minimum Criteria in Massachusetts by MMWR Week, FY2005-2009**

The mean frequency of ILI discharges from 2005-2008 are shown in blue with 95% CIs. Similar to Figure 1, ILI discharges in 2009 fell within the CIs of 2005-2008 until week 17. In 2005-2008, the frequency of discharges with influenza-specific diagnoses declined to approximately zero per week between Weeks 22-39, but we saw a peak of discharges with influenza-specific diagnoses at Week 26 (n=58) during 2009 corresponding to the H1N1 pandemic. This comparison indicates that influenza diagnoses were not common during Weeks 22-39 in years 2005-2008 which is typically non-influenza season, but in the spring and summer of 2009 there was a comparatively large number of discharges with influenza diagnoses from hospitals in Massachusetts. Results in Figure 2 also correspond closely with statewide sentinel site surveillance data from Massachusetts providers reporting percentage of ILI visits [18].

      To address possible misclassification of non-influenza cases included as influenza cases, the breakdown and trends of each code included in the maximum (broad) criteria were analyzed. The H1N1-specific ICD-9 code (488.1) introduced in June 2009 was not used to diagnose any individuals discharged from a hospital in Massachusetts. Among those meeting the maximum criteria, 64% had a discharge code corresponding to pneumonia (486), and 10% had the corresponding discharge code for fever (780.6).

### **Comparing characteristics of H1N1 and seasonal influenza populations**

      Comparing the H1N1 and seasonal influenza groups among the maximum criteria, there were higher proportions of Black, non-Hispanic individuals with H1N1 compared to seasonal influenza (11% versus 9%), as well as Hispanics (12% compared to 10%).  There was also a higher ICU admission rate among those with H1N1 (18% compared to 16%), and statistically significant differences among proportions relating to gender, race/ethnicity, age groups, ICU admission, and ED admission (Table 2). 

      Some of these population differences are more dramatic in the minimum criteria. Compared to seasonal influenza, ILI discharges during the H1N1 pandemic had higher proportions of Black, non-Hispanics (18% compared to 10%), as well as Hispanics (23% compared to 14%). Those with H1N1 were also younger: there were significantly higher proportions of those <18 years with H1N1 compared to seasonal influenza (50% compared to 39%).  Other variables with significantly different proportions between H1N1 and seasonal influenza are ICU admission rates, percentage of those admitted to the hospital through the ED, and those admitted through the outpatient department (Table 2).  

## DISCUSSION

### **Characterization of H1N1-related hospitalizations in Massachusetts**

      We examined influenza cases in Massachusetts using population-based hospital discharge data from HDD. We applied two selection criteria to identify and compare influenza cases. The two study populations are not mutually exclusive since the influenza-specific codes are a subset of the maximum criteria. 

Nearly two-thirds (64%) of those meeting the maximum criteria had a discharge code corresponding to pneumonia. However, studies have found that pneumonia occurred in only 2.5% of those with confirmed H1N1 [23]. One study in particular conducted computed tomography (CT) scans on patients with confirmed H1N1 infection, and discovered that 7.5% (40 of 584 confirmed infections) had chest abnormalities consistent with pneumonia [24]. Since these, and other related assessments, indicate that most of those with H1N1-related symptoms did not have pneumonia during the pandemic, one way to reduce misclassification of those with H1N1 (and possibly other emerging influenza strains) may be to exclude pneumonia from the selection criteria altogether. Including the code for ‘pneumonia’ contributes a large number of cases who may not have confirmed influenza, and with these points in mind, we cannot recommend applying the maximum criteria within an administrative dataset to calculate total influenza caseloads or describe those hospitalized with influenza during a pandemic.

Based on hospital report data between April 25-September 30, 2009, there were 177 people hospitalized in Massachusetts with confirmed H1N1 infection. If we apply the ‘undercount’ calculations [7], this number represents approximately 478 actual hospitalized cases and is closer to the totals found using the minimum criteria (n=601) compared to the maximum criteria (n=10,967). For comparison, we cannot determine precisely how many influenza-related hospitalizations occurred during the same time period in prior years between April and September since influenza-related hospitalizations have no standard reporting protocol, and prior estimates do not exist. Without a population-based data source with reliable estimates of confirmed influenza infection, the estimates provided in this assessment describe a range of possible H1N1-related hospitalizations based on published evidence and methodology. 

Specific to the H1N1 pandemic, hospital discharge coding practice could have changed throughout the duration of the epidemic since increased coverage of the epidemic in the media could influence provider perception, and could influence their diagnoses assigned to patients. To address this, we analyzed individual codes in our maximum selection criteria to discover which were used to flag ILI between April 26- July 15 and July 16-September 30, 2009. Between April 26-July 15, 2009 there were 7,050 total codes used to flag ILI, and 439 (6.2%) were influenza-specific codes. Between July 16-September 30, there were 4,570 total codes used to flag ILI, and 114 influenza-specific codes (2.5%) indicating that the overall use of ILI and influenza-specific codes were used more often in the first time period of the epidemic compared to the second time period. These findings are consistent with another study that reported ILI coding trends during the 2009 H1N1 pandemic [25], also indicating that influenza-specific diagnosis codes were also more likely to be used in the beginning of the pandemic compared to the end, and codes indicating influenza symptoms (such as cough, fever and pneumonia) were used to identify influenza cases more often later in the pandemic. 

Physicians’ perceptions of influenza incidence could have been influenced by media coverage of the magnitude of the epidemic, and could have increased the frequency of diagnoses assigned to patients. However, it is difficult to determine if consistent coding practice occurred throughout the duration of the epidemic. Since influenza-specific codes are the most specific indicator of influenza in administrative data sources using ICD-9 codes, and if they’re going to continue to be used as an accurate indicator of influenza, coding practice should encourage the consistent use of these codes.

**Comparing characteristics of H1N1 and seasonal influenza populations**

One could argue that the smaller, influenza-specific population could be a more accurate and realistic picture of actual influenza activity. Most epidemiological reports described in the literature rely on confirmed hospital case report data to describe H1N1 cases. We have used two selection criteria to describe patients hospitalized with influenza and ILI. Although the methodology of case identification in our study is different than hospital case reporting, we have shown consistent results between published reports and the minimum selection criteria. We found that those who received influenza-specific diagnosis codes overrepresented non-White minority groups, were younger in age, more likely to be male, and had higher rates of ICU admission compared to those who met the more broad criteria. Other characteristics of the influenza-specific group suggest that, of the two criteria used, this group may be a more accurate estimate of actual influenza activity. Those with H1N1 had higher rates of ICU admission compared to those with seasonal influenza. This is consistent with another published assessment restricted to a metropolitan area in the United States [13]. Also shown in the literature, H1N1 affected all age groups, but frequently occurred in previously healthy, young adults with a wide range of socio-demographic characteristics and clinical presentations [26]. In the Northern Hemisphere, hospitalized patients with confirmed or probable H1N1 infection ranged in age from 21 days to 86 years, but 45% of hospitalized cases were under the age of 18 years, and only 5% of cases were 65 years of age or older [27]. State reports based on laboratory-confirmed H1N1 data indicate that 45% were <19 years, 33% 19-49 years, 15% 50-64 years, and 7% ≥65 years [19]. This is consistent with what we found in the minimum criteria (50% were <18 years), but not the maximum (23% were <18 years, and 51% were aged 45-64 years). In fact, the maximum criteria showed a greater distribution of 45-64 year olds during the H1N1 pandemic compared to prior seasonal influenza seasons. 

Similar trends existed in relation to race/ethnic group characteristics. Unpublished statewide 2009 H1N1 epidemiological data suggests that there have been disproportionate numbers of laboratory-confirmed and hospitalized H1N1 cases among certain racial/ethnic groups in Massachusetts. The Massachusetts DPH found that rates of lab-confirmed H1N1-related hospitalization were three to four times higher in Black and Hispanic populations compared to White, non-Hispanic populations (Alfred DeMaria, MDPH, personal communication, 2010). Furthermore, the CDC reports greater proportions of hospitalizations and deaths among particular ethnic groups in Chicago [28] and among American Indian/Alaskan Natives compared to other racial groups [29]. From our analyses, we can see that both H1N1 selection criteria have higher proportions of Black and Hispanic groups compared to seasonal influenza. However, proportions of non-White race/ethnic groups among those in the narrow criteria (47% White, non-Hispanic) correspond more closely to rates in published epidemiological reports compared to broad criteria (70% White, non-Hispanic).

From the results described, it becomes clear that although the maximum criteria have been used to describe influenza activity and have correlated with confirmed influenza in prior studies [20] [25], these selection criteria may be too non-specific for the purposes of influenza case detection among those hospitalized with H1N1. The maximum criteria utilized in this study display high frequencies of ILI-related discharges – even during summer months, which is typically outside of the influenza season (Figure 1). Furthermore, those included in the maximum criteria do not correspond with epidemiological descriptions presented in the literature, the total caseload between Apr-Sept 2009 (n=10,976) could be a gross overrepresentation of actual hospitalized cases, and since additional respiratory syndromes are included in the maximum selection criteria, misclassification due to non-influenza is not a possibility - it is a certainty. These results correspond with another recent assessment which found that these same criteria did not sufficiently monitor/detect the H1N1 outbreak, had inadequate sensitivity, and poor positive predictive value for identifying ILI cases [30]. 

However, there are major limitations to using the minimum criteria as an indicator for disease activity. Assigning influenza-specific codes to patients in administrative data sources is not dependent on confirmed influenza test results, and there is no way, other than manual chart review, to confirm if someone who received an influenza diagnosis actually had the disease. We were authorized to use this dataset only in a de-identified way, and manual chart review was therefore not feasible. Studies investigating the use of ICD-9 codes to identify influenza cases have found that confirmed-positive pandemic 2009 H1N1 cases were not uniformly coded as influenza, and nearly 10% of confirmed cases were not even captured in a syndromic surveillance ILI algorithm [25] further suggesting that there are many difficulties detecting and tracking confirmed influenza cases using administrative data sources. Finally, there is a lack of standard clinical protocol for coding influenza diagnoses, and it is difficult to understand the process of clinical decision-making related to influenza diagnoses by analyzing ICD-9 codes. 

**Limitations**

Data from correctional facilities and veteran’s hospitals in Massachusetts are not included in the HDD, and there is also limited information related to treatments and diagnoses in emergency departments. Reports of those with self-reported ILI symptoms during the H1N1 pandemic in the U.S. indicate that 40% of adults and 56% of children sought health care [31]. Because the HDD only includes those hospitalized with H1N1, we were not able to compare this hospitalized population to non-hospitalized H1N1 cases to see differences in demographics, diagnoses, or outcomes. 

CONCLUSIONS

This is the first population-based assessment of epidemiological characteristics of hospitalized H1N1 cases in Massachusetts, and it is the first to include all possible hospitalized cases in the analysis. We found that large administrative data sets, like HDD, can be used to detect hospitalizations for influenza during a pandemic, but estimated case counts vary widely depending on the selection criteria used.  We compared selection criteria currently in use for surveillance and case detections (maximum criteria) with influenza-specific ICD-9 diagnosis codes (minimum criteria), and have provided a range of H1N1-related hospitalizations. In this study, the maximum criteria overestimated H1N1 activity, and we do not recommend using these criteria to characterize the epidemiology of influenza study populations. Those meeting minimum criteria most closely resemble published descriptions of H1N1-related hospitalizations and meet the expected influenza case count, and we recommend using the minimum criteria to describe populations hospitalized with influenza, despite the limitations noted above. We can confirm that H1N1 affected a younger population, and disproportionately affected racial minorities in Massachusetts. There were also higher rates of ICU admission compared to seasonal influenza. 

Reporting population-level rates of disease, however, is challenging without large databases containing confirmed disease indicators. Improving the quality of population-based data sources with reliable indicators of influenza could provide reasonable indicators of disease. For now, however, one of the best ways to report disease activity in the U.S. is through ICD-9 codes, which may not necessarily reflect care given or individual symptoms while in the hospital. With this in mind, future work should establish a ‘best practice’ for influenza case identification within administrative data sources to more accurately identify and describe population-based rates of disease, severity, and mortality. 

**Acknowledgments:**

The authors would like to acknowledge the following individuals for their contribution to this manuscript:  Bruce Barton, PhD (UMMS), John Brownstein, PhD (CHIP), Bruce Cohen, PhD (MDPH), James West, PhD (MDPH), Alan Rothman, MD (URI), Richard Ellison, MD (UMMS), and Brian O’Sullivan, MD (UMMS). 

**Funding information:**

We would like to acknowledge the support of Massachusetts Department of Public Health, Division of Epidemiology and Immunization and Clinical and Population Health Research Department at University of Massachusetts Medical School.

**Competing interests:**

The authors have declared that no competing interests exist. 
